# Visualization of human optic nerve by diffusion tensor mapping and degree of neuropathy

**DOI:** 10.1371/journal.pone.0278987

**Published:** 2022-12-12

**Authors:** Łukasz Łabieniec, Łukasz Lisowski, Horia I. Petrache, Marcin Hładuński, Joanna Konopińska, Jan Kochanowicz, Krzysztof R. Szymański

**Affiliations:** 1 Department of Condensed Matter Physics, University of Bialystok, Bialystok, Poland; 2 Department of Ophthalmology, Medical University of Bialystok, Bialystok, Poland; 3 Department of Physics, Indiana University Purdue University Indianapolis, Indianapolis, IN, United States of America; 4 Independent Laboratory of Molecular Imaging, Medical University of Bialystok, Bialystok, Poland; 5 Department of Neurology, Medical University of Bialystok, Bialystok, Poland; Henry Ford Health System, UNITED STATES

## Abstract

Diffusion-weighted magnetic resonance imaging of the human optic nerve and tract is technically difficult because of its small size, the inherent strong signal generated by the surrounding fat and the cerebrospinal fluid, and due to eddy current-induced distortions and subject movement artifacts. The effects of the bone canal through which the optic nerve passes, and the proximity of blood vessels, muscles and tendons are generally unknown. Also, the limited technical capabilities of the scanners and the minimization of acquisition times result in poor quality diffusion-weighted images. It is challenging for current tractography methods to accurately track optic pathway fibers that correspond to known anatomy. Despite these technical limitations and low image resolution, here we show how to visualize the optic nerve and tract and quantify nerve atrophy. Our visualization method based on the analysis of the diffusion tensor shows marked differences between a healthy male subject and a male subject with progressive optic nerve neuropathy. These differences coincide with diffusion scalar metrics and are not visible on standard morphological images. A quantification of the degree of optic nerve atrophy in a systematic way is provided and it is tested on 9 subjects from the Human Connectome Project.

## Introduction

In biological tissues, water translational diffusion is influenced by microstructural components, including cell membranes and organelles. The optic nerve is an almost pure white matter tract that has an intrinsically high water restricted diffusion due to its highly packed axons and the encompassing nerve sheaths [1]. The movement of water molecules can be measured by magnetic resonance imaging (MRI). The most advanced MRI technique proposed in 1994 [2] allows the measurement of directional diffusion and is called diffusion tensor imaging (DTI) [3–7]. This method comprises a group of techniques in which the eigenvalues and eigenvectors of the diffusion tensor are used to create images reflecting various diffusion properties of a tissue [8–10]. DTI can provide information on anatomical connectivity in the brain by measuring the anisotropic diffusion of water in white matter tracts [11–20] or in gray matter structures [21]. One of the most commonly used quantitative measures of the tissue microstructures is the fractional anisotropy (*FA*) [22], indicating how strong the directional diffusion in the local voxel is. Many imaging studies are starting to use *FA* images from multiple subjects in voxelwise tract-based spatial statistical analyses, in order to localize brain changes related to development, training, degeneration or disease, and to explore anatomical connectivity in the brain and compare with other nonhuman primates [23–28]. In this method, called tractography, "seed voxels" are selected in a certain area of the brain, and then fiber trajectories are computed by an automated software.

Tractography is a method for identifying white matter pathways in the living human brain and it is the only available tool for non-invasive and *in vivo* identification and measurement of these pathways. Although many studies have shown tractography to be of promising value for neurosurgical care [29, 30], some results remain inconclusive [31]. The idea of fiber tracking is to follow the dominating eigenvector from the selected seed voxel (or group of voxels) to encounter neighboring voxels, at which the trajectory is changed according to the direction of the new dominating eigenvector in the neighboring voxel. Fiber tracking ends when one of the following conditions is met: 1) the tract reaches the boundary of the imaging volume, 2) the tract reaches a region with low diffusion anisotropy, 3) the radius of curvature of the tract is smaller than a selected threshold radius, or 4) the most collinear eigenvector is not associated with the largest eigenvalue [32]. The resulting trajectories can be used as a region of interest for *FA* and other scalar parameters voxel-based measurements.

In principle, the DTI technique should be helpful in visualizing the optic nerve and tract. Unfortunately, because of the insufficient spatial resolution of the images, the optic nerve, as well as spinal cord, are technically difficult to investigate with any MRI technique, especially DTI [33]. It is challenging for current tractography methods to accurately track optic pathway fibers that correspond to known anatomy. However, systematic progress is being reported [34] and several studies have shown successful tracking of some parts of the optic pathway [35–39]. Examples of visualization of retinogeniculate visual pathway fiber displacement due to tumors have been reported in [37, 38, 40].

**Fig 1:**
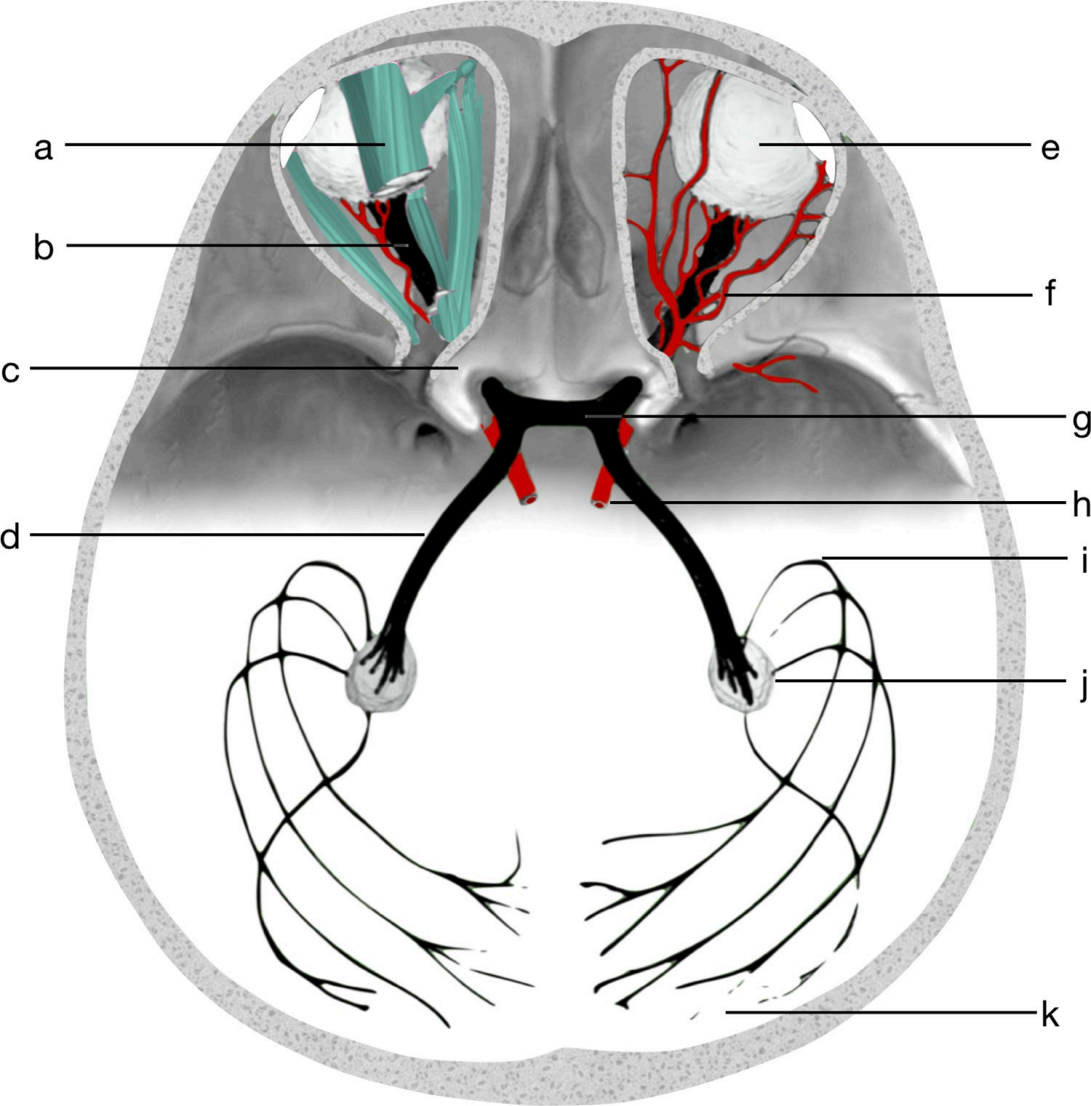
A schematic anatomical overview of optic pathway and surroundings, a–muscles and tendons, b–optic nerve, c–nearby bony structures (optic canal), d–optic tract, e–eyeball, f–blood vessels, g–optic chiasm, h–ophthalmic artery, i–Meyer’s loop, j–lateral geniculate nucleus, k–optic radiation and visual cortex.

The optic nerve is surrounded by cerebrospinal fluid and fat, which significantly affect the measured signal. It is also not known what the effect of the presence of nearby bony structures, veins, or even muscles and tendons is (Fig 1). An abnormal signal near the optic canal (Fig 1C), where the optic nerve is surrounded by bone and air from nasal sinuses, results in negative diffusion tensor eigenvalues [34]. The small size of the optic nerve and its movement during scanning make it even harder to obtain high-quality images. Additionally, sequences such as planar imaging (EPI) used in DTI are more sensitive to image artifacts and eddy current distortions than any other MRI technique [41–43]. Limitations of the EPI include low spatial resolution, blurring effects of T2 decay occurring during image readout, and sensitivity to artifacts due to Nyquist ghosting, chemical shift, magnetic field inhomogeneity, and local susceptibility effects [6, 44]. Although it is possible to obtain a high-resolution tractography of the optic pathway in animals, the acquisition time is several dozen hours [45] or even few days [46]. Owing to the technical limitations of the current scanners and long acquisition time requirements, it is impossible to obtain good-quality diffusion images of the optic pathway and hence probabilistic tractography results. The curvature of the optic nerve is very high. None of the tractography methods are inherently able to properly deal with complicated topology and with the intricate morphology of crossing and branching fibers. Scalar diffusion parameters of the optic nerve are much more often measured by manual selection of the area of interest by qualified radiologists or from tracts found with tractography, manually trimmed by an experienced physician to better match the anatomy [47].

Several studies have found strong correlations between the severity of glaucoma and a reduction in *FA*, as well as an increase in mean diffusivity (*MD*) in the optic nerve [48–51]. Patients with optic neuritis have been found to have increased *MD*, and decreased *FA* [52], with similar findings reported in patients with multiple sclerosis [53]. Higher *MD* have been found in retinitis pigmentosa than in healthy controls, as well as lower *FA* [54]. The amblyopic patients showed significant reductions in optic nerve and optic tract microstructural integrity with decreasing *FA* in the optic nerve and optic tract [47]. However, because of the low resolution of the diffusion images, the results are often inconclusive, and correlations are not sufficiently strong. Because of this, is it difficult to assess the exact degree of the optic nerve neuropathy. In some cases [55], for example in optic neuritis [56] or glaucoma [57], the optic nerve can be assessed from morphological images based on T1 or T2.

In the standard DTI visualization, the principal component of the diffusion tensor at each voxel is displayed with line segments [58]. It is also possible to display multiple line segments per voxel that show the second and the third eigenvectors. For color coding of displayed line segments, standard RGB convention is used where the red, green and blue colors represent diffusion along the *x-*, *y-* and *z*-axes, respectively. Three eigenvectors can also be visualized in the form of an ellipsoid with symmetry axes parallel to the eigenvectors and the color depending on the dominating eigenvalue [4, 7, 59]. With our algorithm, we significantly improve the visibility of the optic nerve, chiasm and tract by an analysis of eigenvectors and eigenvalues and an optimized assignment of visual components that include segments length, opacity and color depth which together enhance the visibility of the fiber structures.

## Materials and methods

This study was performed in compliance with the Declaration of Helsinki and was approved by the local ethics commission (Medical University of Bialystok under the number R-I-002/477/2019). Written and informed consent was obtained from all subjects prior to study inclusion (ClinicalTrials.gov Identifier: NCT05353413).

### Study design

In the present study, images from a healthy male subject (age: 32) and a male subject with penetrating right eyeball injury (age: 46) were used. Both subjects underwent a full ophthalmological examination, which included visual acuity, color vision, applanation measurement of intraocular pressure, evaluation of the anterior chamber and the fundus of the eye using a slit lamp and the Volk lens, optical coherence tomography examination of the macula and optic nerve disc (OCT), neurological field vision, and visual evoked potential (VEP) testing. In the tests conducted, no deviations from the normal condition were found in the subjects, while the trauma patient had endotamponade of the vitreous chamber of the right eye with silicone oil, no light perception, secondary glaucoma, and atrophy of the right eye optic nerve, and correct ophthalmological examination of the left eye. In his medical history, the patient reported that prior to the penetrating injury (metal shard from a masonry hammer driven into the eyeball), the patient did not experience any deterioration in vision compared to the left eye. During active scanning, the patients tried not to blink and did not move their heads, while gazing at the red-locking LED outside the camera reflected in the mirror of the coil.

### Diffusion tensor imaging

MRI data were acquired using a Biograph mMR 3-Tesla scanner (Siemens, Erlangen, Germany) with a 16-channel head-neck coil. Acquisition parameters were set up based on the [60] and technician experience. The total duration of the EPI DTI examination was 10:34 min (8:05 and 2:29 for A>>P and P>>A phase encoding directions respectively). MRI acquisitions were obtained using a scanner with a maximum gradient amplitude of 45 mT/m and a maximum slew rate of 200 T/m/s. Parameters for used for echo planar (EPI) acquisition method were: TR 7000 ms, TE 113 ms, echo spacing 0.97 ms, bandwidth 1184 Hz/Px, EPI factor 128, voxel size 1.3×1.3×2.0 mm, 25 slices (transverse orientation), distance factor 0% for transverse plane and 50% for other planes, base resolution 128 and prescan normalization filtering. Sixty-four non-collinear diffusion directions for a *b* value of 1000 s/mm^2^ with one average were acquired for the A>>P encoding direction and six averages without diffusion-weighted acquisition for *b* value of 0 s/mm^2^ were acquired for both the A>>P and the P>>A encoding directions. Parallel imaging with an acceleration factor of 2 was enabled using the GRAPPA algorithm. Additionally, an anatomical T1-weighted (MP RAGE), scan time 5:21 min, voxel size 1.0×1.0×1.2 mm, was performed.

In addition to coronal magnetization-prepared rapid acquisition gradient-echo reconstruction, oblique transverse sections through both optic nerves were done. The exact positions and angulation of slices were further graphically specified and adjusted in the central sections of the left and right sagittal optic nerves. This procedure allowed for an exact adjustment of the EPI and T1 slices through both optic nerves. In general, positioning was focused on the part of the optic nerve closest to the eye to allow for optimal comparability in the case of a curved optic nerve.

### Image post-processing

Diffusion images were converted from the DICOM format to the NIfTI format using the open source *dcm2niix* software [61], and the data were post-processed using the FSL software library (http://www.fmrib.ox.ac.uk/fsl) [62]. The ITK-SNAP software (http://www.itksnap.org) was used to create a binary mask of the brain, eyeballs, and optic nerves. FSL’s tools were used for susceptibility -induced and eddy current -induced distortion correction, and movement artifacts correction [63–65]. The correction procedure assumes that the eddy current-induced field can be modelled as a combination of linear and quadratic terms. Calculations were parallelized with CUDA v9.1 on Nvidia Tesla K80 GPU. Computations were carried out at the Computer Center of University of Bialystok, Poland. Eddy current and motion-corrected diffusion-weighted images were used for voxel-by-voxel-based tensor calculations. Consequently, eigenvectors (*V*_1_, *V*_2_ and *V*_3_) and eigenvalues (*L*_1_≥*L*_2_≥*L*_3_) were obtained. Throughout the text, the largest eigenvalue *L*_1_ will be called axial diffusivity. For all other calculations, the Wolfram Mathematica software was used and NIfTI images were imported using the QMRITools toolbox developed for the Wolfram language [66]. Fractional anisotropy (*FA*), mean diffusivity (*MD*) and radial diffusivity (*RD*) were defined as in [7, 44, 60]:

$$FA=\frac{{\left(L_{1}-L_{2}\right)}^{2}+{\left(L_{2}-L_{3}\right)}^{2}+{\left(L_{1}-L_{3}\right)}^{2}}{2(L_{1}^{1}+L_{2}^{2}+L_{3}^{2})},$$ (1)

$$MD=(L_{1}+L_{2}+L_{3})/3,$$ (2)

$$RD=(L_{2}+L_{3})/2.$$ (3)

### Algorithm

**Fig 2:**
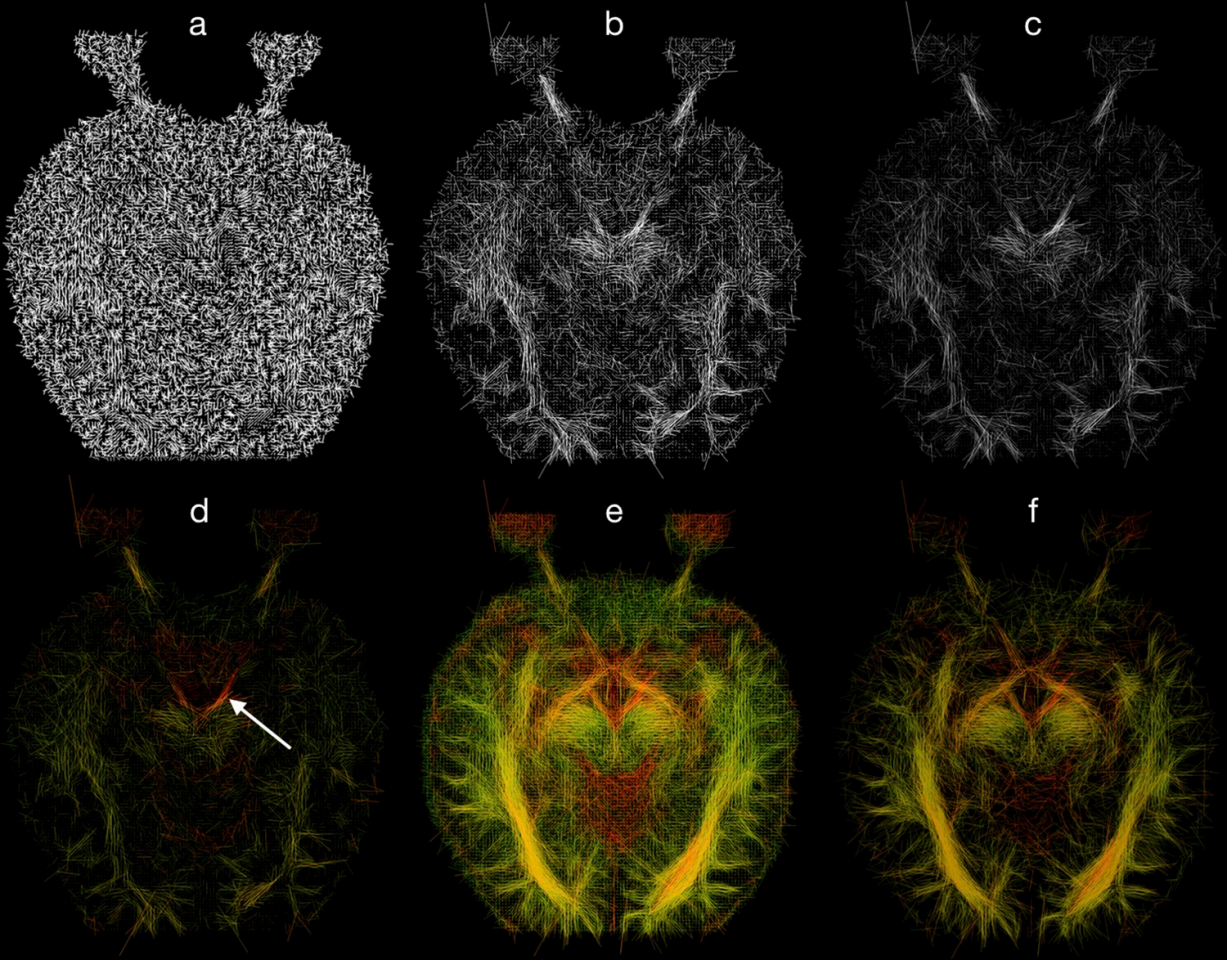
The visual effect of the algorithm in six steps for a healthy subject, a–principal diffusion directions shown as line segments, b–length coding, c–opacity coding, d–color coding, e–seven layers projection, f–seven layers projection and data thresholding. In a-d a single layer was used. White arrow: oculomotor nerve. Orientation convention: Radiological.

The physical property of the diffusion tensor is that all eigenvalues are non-negative. However, correction algorithms and low-resolution data can lead to negative eigenvalues that are clearly non-physical. Therefore, in our procedure all negative, nonphysical eigenvalues were set to zero [67]. The data visualization algorithm consisted of six steps each having a significant impact on the displayed image. The visual effect accomplished by each step is presented in Fig 2A–2F and described below.

In the first step, the first eigenvector *V*_1_ in each voxel is displayed as a line segment representing the main direction of diffusion in that voxel, and the line segments are given the same length (Fig 2A). Because the morphological structure of the optic nerve favors the presence of one particularly distinguished direction of diffusion, we should focus especially on voxels in which diffusion is strongly directional *i*.*e*., *L*_1_≫*L*_2_. Hence, in the second step, the length of the line segment in each voxel is adjusted to a value given by

$$\alpha {\left(L_{1}-RD\right)}^{2},$$ (4)

where α is a scaling parameter. This means that the more *L*_1_ deviates from *RD*, the longer the line segment for that voxel (Fig 2B).

Another important piece of information is the strength of diffusion in each voxel. Thus, in the third step, the opacity of the line segment in each voxel is set proportional to the product of the mean diffusivity and fractional anisotropy,

β(MD∙FA), (5)

where *β* is a scaling parameter. This means that the stronger the diffusion, the opaquer the segment for that voxel (Fig 2C).

The 4th step in our algorithm is an RGB color assignment that improves image clarity based on *L*_1_ values as follows

$$Red={\gamma L}_{1N},$$

$$Green=1-L_{1N},$$ (6)

Blue=0,

where *γ* is a scaling parameter for setting the intensity of red color, and *L*_1*N*_ is a normalized value, *L*_1*N*_ = *L*_1_/*L*_1*max*_, where *L*_1*max*_ is the maximal value among all voxels *L*_1_ (Fig 2D).

**Fig 3:**
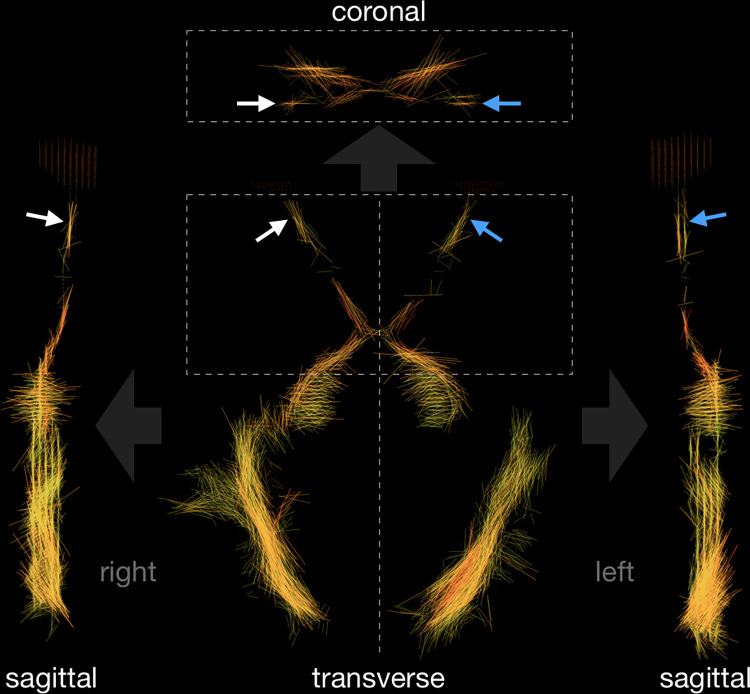
Visual pathway shape on different plane projections. White and blue arrows indicate right and left optic nerve, respectively. Real voxel proportions are preserved.

The goal of choosing a different color scaling than in the standard tractography is to assume that the *L*_1_ values should be similar along the entire length of the optic pathway, and therefore, it should be presented in a similar color. Furthermore, standard tractography color coding appears to be ineffective for the optic pathway because this structure does not form a straight-line segment in three-dimensional projections and such coding would unnecessarily introduce different colors on different sections of the optic nerve and tract. For this reason, for optic pathway visualization, more than one layer must be used. Therefore, we propose a new approach. In step five, we projected the three-dimensional shape of the visual pathways onto the transverse plane (Fig 2E). Consequently, although the information about shape in the direction of the projection is lost, we gain a much-improved visibility in the two other directions (*i*.*e*., the plane perpendicular to the projection direction). On the transverse plane, the visual pathway is best visualized, but to show the complete location of the nerve, we project manually the segmented optic pathway onto the sagittal and coronal planes (Fig 3). After the projection of a few layers onto one plane, we obtain a superposition of line segments in each voxel, producing enhanced visibility. However, segment superposition can affect image clarity and for this reason we implement a 6th step in which short line segments are discarded based on the following cutoff condition

$$L_{1N}>{RD}_{N}+\sigma ,$$ (7)

where *σ* is a threshold value (Fig 5), *L*_1*N*_ and *RD*_*N*_ are the normalized values of the *L*_1_ and *RD* respectively. The result is that line segments are displayed only if the axial diffusivity exceeds the radial diffusivity by more than a chosen value for *σ* (Fig 2F).

The presented algorithm and projections in three directions are particularly suited for the imaging of fiber structures. The optic nerves are clearly visualized in Fig 3 (arrows). The right-hand side nerve is visualized as a single fiber, while the left-hand side nerve is split on coronal and sagittal projections because it is located just between the two planes used in the imaging protocol. Because the imaging protocol includes pre-scan normalization filtering, the parameters *α*, *β*, *γ* and *σ* were selected once to ensure the best visibility of the visual pathways. These parameters may require modifications if any changes in the protocol arise or when different scanner devices are used. An unexpected but useful result is that the parameter *σ* can serve as a quantitative measure in the diagnostics of nerve atrophy as shown later in the manuscript.

### Estimation of a threshold value of the *σ* parameter

The threshold value of the *σ* is estimated by Formula (7). It is the maximal value of the *σ* for which condition (7) is true for all pixels within the region of interest. In another words, the threshold value of the *σ* is equal to the largest difference between axial and radial diffusivity for the pixels inside the region of interest. A boundary of the region of interest includes an optic nerve ranging from eyeball to the optic canal.

### Image quality testing

Diffusion tensor estimation can produce indefinite diffusion tensors that arise in locations where the diffusion-weighted signal is disturbed by noise and the degree of anisotropy is high [68]. In biological tissue, all eigenvalues of the diffusion tensor are assumed to be positive but due to noise or signal drop, negative eigenvalues may be generated [67]. The fraction of voxels for which negative eigenvalues have been generated relative to the total number of all voxels, expressed as a percentage, can be used as a measure of image quality. For a healthy subject, those values were 0.61%, 6.5% and 9.0% for *L*_1_, *L*_2_ and *L*_3_ maps, respectively. In the case of a subject with a damaged eyeball, these values reached the values of 0.99%, 2.3%, and 9.5%, respectively.

### Other non-atrophic optic nerves

Datasets from nine healthy subjects (the “Young Adult Diffusion Dataset” release of healthy adults between the ages of 20 and 59 [69]) from the HCP database (https://www.humanconnectome.org) [70] were used for experimental evaluation. The HCP data scanning protocol was approved by the local Institutional Review Board (IRB) at Washington University. The HCP database provides diffusion data that was acquired with a high-quality image acquisition protocol using a customized Connectome Siemens Skyra scanner and processed using a well-designed processing pipeline [71] including motion correction, eddy current correction and distortion correction. The diffusion acquisition parameters in HCP were: TR = 5.520 ms, TE = 89.5 ms, FA = 78°, voxel size = 1.25 × 1.25 × 1.25 mm, and FOV = 210 × 180 mm. A total of 288 images were acquired in each diffusion dataset, including 18 baseline images with a low diffusion weighting *b* = 5 s/mm^2^ and 270 diffusion weighted images evenly distributed at three shells of *b* = 1,000/2,000/3,000 s/mm^2^. In this study only single-shell *b* = 1,000 s/mm^2^ data, consisting of 90 diffusion weighted images and 18 baseline images, were used for calculating with our algorithm. Single-shell *b* = 1,000 s/mm^2^ data were used because it is similar to our clinical acquisition protocol. Furthermore, single-shell *b* = 1,000 s/mm^2^ data has been shown to be more effective for identification of cranial nerves than higher *b* values [72]. A visual check of the diffusion data for each of the subject was performed and any subjects whose diffusion data had incomplete optic nerve coverage were excluded. An FSL Nudge Tool was used to manually adjust the affine for every image to cover the layers along the optic nerve and then the diffusion tensor was calculated. Finally, nine subjects were chosen, and thresholds of *σ* value were calculated.

## Results

**Fig 4:**
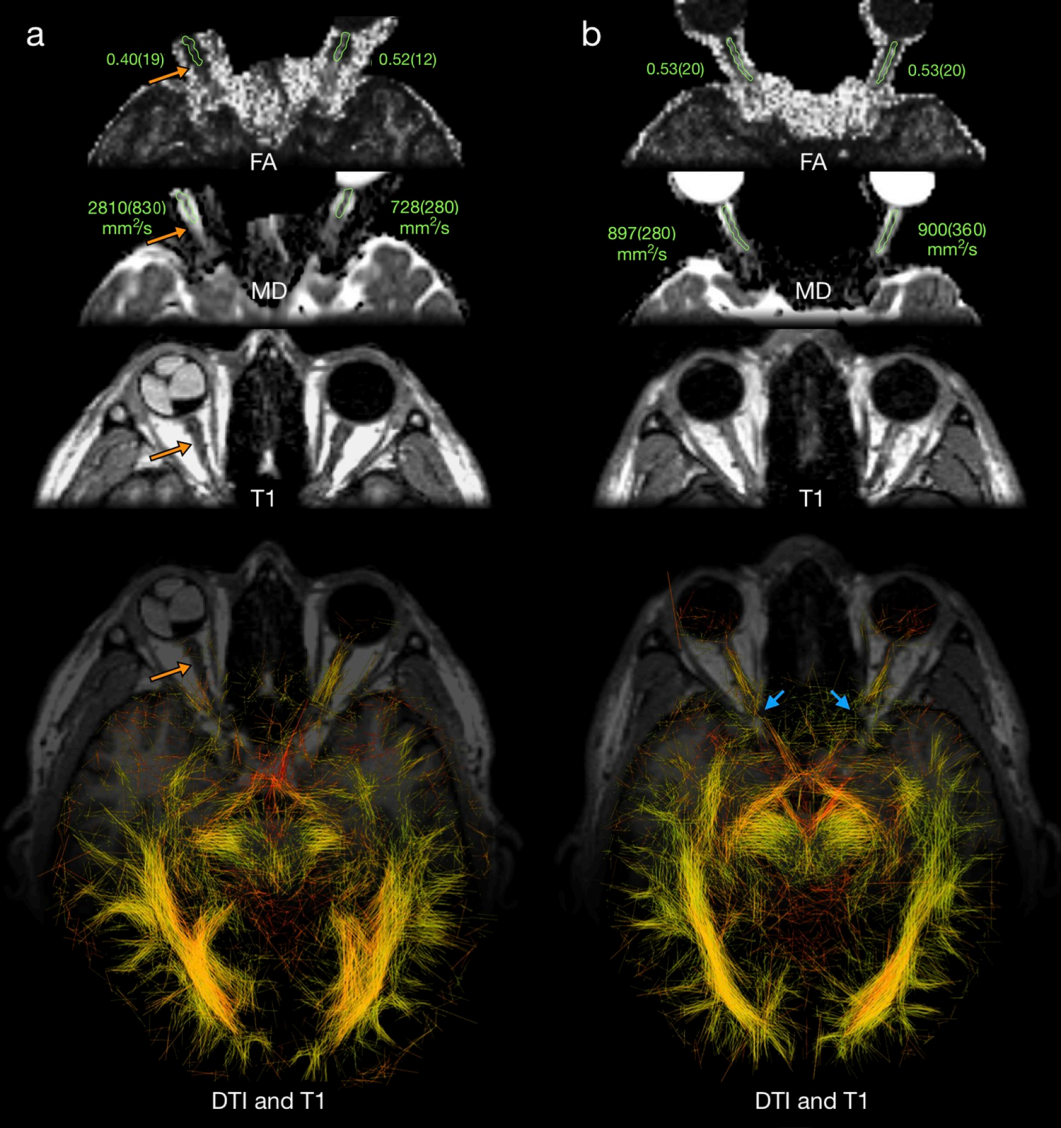
Visual pathway of male subject with right optic nerve atrophy (orange arrows) (a) and for healthy male subject (b) visualized with the new DTI algorithm superimposed on the same T1 image. Optic nerves are also presented on diffusion scalar maps FA and MD, and on morphological T1-weighted images. Actual values of FA and MD measured in a region of interest containing the optic nerve are presented in green. Blue arrows: optic canal and locations of visible discontinuities. Orientation convention: radiological. Values of *α*, *β*, *γ* and *σ* used in visualization were 250, 1000, 1.2 and 0.2, respectively.

The presented algorithm was used to generate images of the visual pathway in a healthy male subject (Fig 4B) and a male subject with the right optic nerve atrophy caused by eyeball injury (Fig 4A). For progressive optic neuropathy in the second subject an increased MD and decreased FA in the optic nerve have been found, compared to the healthy optic nerve of the same subject, and compared to the healthy optic nerve od the first subject (Table 1). In T1 image a constriction of the atrophic nerve compared to the left optic nerve have been found (Fig 4). The DTI image represented as line segments generated by the algorithm presented in this work showed significant difference between the atrophic and fully functional optic nerves with no segmentation required.

**Table 1:** Fractional anisotropy (FA), mean diffusivity (MD) in the optic nerve and *σ* threshold value for with the optic nerve is removed from the image.

	subject with penetrating right eyeball injury		healthy subject	
	right eye	left eye	right eye	left eye
FA	0.40(19)	0.52(12)	0.53(20)	0.53(20)
MD [mm^2^/s]	2810(830)	728(280)	897(280)	900(360)
*σ*	0	0.35	0.30	0.36

**Fig 5:**
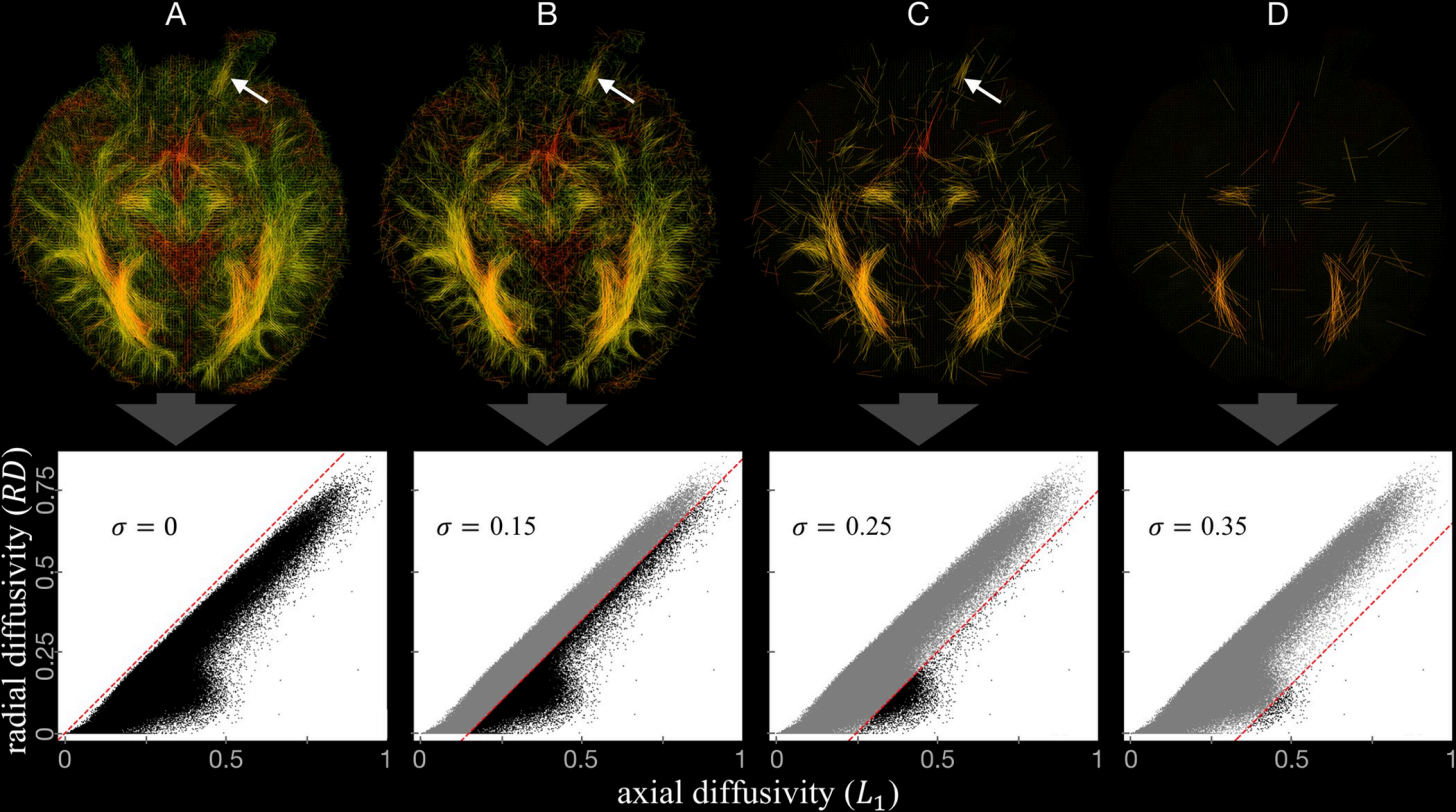
Images for male subject with right optic nerve neuropathy, generated for *σ* values equal to A) 0, B) 0.15, C) 0.25, and D) 0.35. Density plots in the bottom panels show all voxels in the datasets used for visualization (black dots) and voxels omitted according to condition (7) (grey dots). The dots are obtained from all voxels in the selected layers. The red dashed lines correspond to the condition *L*_1*N*_ = *RD*_*N*_+*σ*. The left optic nerve (white arrow) is visible (A,B,C) until a certain value of *σ* is exceeded (D). Orientation convention: Radiological.

The most important for unidirectional diffusion data were extracted by introduced threshold parameter *σ*. A set of images generated for different values of *σ* show that this parameter can be used as an indicator of the degree of neuropathy (Fig 5). Both optic nerves from a healthy subject and left optic nerve from the subject with right eyeball injury are removed from the image when *σ* exceeds almost the same thresholding level (Table 1). In contrast, the atrophic nerve is not visible even for *σ* equal to zero (Fig 5A). An example of the region of interest used for determining the *σ* value is shown in green in Fig 4.

Threshold values of *σ* for which the optic nerve is removed from the image, calculated for 21 non-atrophic optic nerve (every nerve from 9 HCP subjects, two nerves from healthy subject, and one healthy nerve from subject with right eyeball injury) were similar (Table 2).

**Table 2:** Distribution of *σ* value for 21 non-atrophic optic nerves.

parameter	value
median	0.33
mean	0.33
standard deviation	0.03
min	0.27
max	0.39

It is important to note that the presented algorithm allows visualization of the oculomotor nerve (indicated by the white arrow in Fig 2D), consistent with the anatomical atlases [73–75]. The bone canal through which the optic nerve passes (optic canal) may be the reason for the observed optic nerve discontinuities at this point (marked with blue arrows in Fig 4).

## Discussion

Visualization of the optic nerve is necessary because in many cases where patients report visual impairment, changes are seen on electroencephalography but not on MRI images. The electroencephalographic result itself is sometimes ambiguous and in order to make an accurate diagnosis, it is necessary to compare it with another method. Apart from clinical and electrophysiological evaluation, MRI plays an important role in the complete assessment of optic nerve and the entire visual pathway. MRI images are helpful in describing the segmental anatomy of the optic nerve and to findings of various conditions affecting the optic nerves [55]. Because of the insufficient spatial resolution of the diffusion MRI images, it is difficult to assess the degree of the optic nerve neuropathy.

The presented algorithm showed the possibility of a direct visualization of data for the optic nerve by diffusion tensor mapping, without relying on probabilistic imaging tools. We have shown that appropriate matching and combining scalar values of the diffusion tensor with the eigenvector data can result in the generation of an image that shows a significant advantage in optic nerve neuropathy assessment. The atrophic and healthy nerves can be differentiated with our algorithm without any segmentation and can be correlated with diffusion voxel-based metrics such as FA or MD.

Decreased FA and increased MD found in the optic nerve with progressive optic neuropathy coincide with the values reported for amblyopathy, glaucoma, optic neuritis, retinitis pigmentosa and multiple sclerosis [47–54, 57].

We have shown that the projection of a few layers on a single plane is a preferential method to view fibrous tissues. Even though some 3D information is lost, the visibility enhancement of the entire structure is significant. Since multilayer projections cannot be merged with single layer morphological images due to possible mismatch, in Fig 4 we show only a part of the T1 image in the background.

In diffusion tensor imaging, the subject uncontrolled blinking, eyeball movements, and spatial and temporal variations of the static magnetic field caused by susceptibility effects and time-varying eddy currents result in severe distortions, blurring, and misregistration artifacts [63–65, 76]. Due to the limitations of acquisition time and attempts to shorten it to a minimum for the patient’s comfort, only the part of the head containing the optic nerve and the tract was scanned.

It is important for our protocol that the orientation of layers is chosen such that they are parallel to the optic nerve axis to a large extent, which is different than standard brain scanning.

By finding the *σ* threshold value for which the optic nerve is removed from the image, we obtain a quantitative measure of optic nerve neuropathy. Other numerical voxel-based measurements are possible by optic nerve segmentation from images in which voxel brightness intensities are set based on the product of segment length and opacity. In such a monochrome image, it is easy to draw a region of interest’s mask, for example, on the part of the optic nerve starting behind the eyeball and ending on the skull optic canal, where the line continuity is lost. Founded masks can be automatically corrected by *σ* parameter, which may be a threshold value used for semi-automatic segmentation [77], or active contour segmentation [78]. Both methods can be alternatives to different tractography segmentations [34] and manual methods.

Nine subjects from HCP database were not underwent a full ophthalmological examination, and if there are any visual diseases, this may be the reason of the *σ* values fluctuations. No visual fixation and eyeballs movement during the examination made it difficult to distinguish the optic nerve from the periocular muscles. The data presented in Table 2 show that standard deviation of the *σ* parameter is over ten times smaller than the average value confirming reasonably good reproducibility.

It was checked that the particular choice of parameters *α*, *β* and *γ* (Eqs (4), (5), (6) and Fig 4) that resulted in improvement of nerve visualization does not affect the threshold value of *σ* at which the optic nerve is removed from the image (Fig 5).

As with most studies, our study faces certain limitations. Our imaging acquisition requires 10 minutes of gaze fixation, which may be challenging for patients who require sedation or are unable to fix their gaze. Our study is also limited to a single case of abnormal vision due to trauma causing blindness and optic nerve atrophy. The application of this technique to patients with less severe vision loss as well as comparison to other techniques is planned for future studies. Finally, the use of isotropic voxels and a higher channel head coil may further improve our diffusion technique. However, despite these limitations, we feel that the described approach may be a helpful complement to existing tractography methods of the optic nerve.

## Conclusions

The proposed new approach of diffusion tensor visualization of the optic nerve and tract can be a complementary to the standard tractography methods, particularly in cases when the standard results that do not fit well with the known anatomy. Our method properly accounts for fiber curvature without relying on probabilistic imaging tools. The images generated by the algorithm presented in this study clearly show significant differences between the atrophic and healthy optic nerves from the eyeball to the optic chiasm and the *σ* parameter in our algorithm provides a quantitative measure of nerve atrophy. The estimation of the parameter on 21 non-atrophic optic nerve show reasonably good reproducibility since the standard deviation is ten times smaller than its average value.
